# Compatibility and Efficacy of the Parasitoid *Eretmocerus hayati* and the Entomopathogenic Fungus *Cordyceps javanica* for Biological Control of Whitefly *Bemisia tabaci*

**DOI:** 10.3390/insects10120425

**Published:** 2019-11-25

**Authors:** Da Ou, Li-Mei Ren, Yuan -Liu, Shaukat Ali, Xing-Min Wang, Muhammad Z. Ahmed, Bao-Li Qiu

**Affiliations:** 1 Key Laboratory of Bio-Pesticide Innovation and Application, Guangdong province, Guangzhou 510640, China; ouda_IPM@163.com (D.O.); LYuan@stu.scau.edu.cn (Y.-L.); aliscau@scau.edu.cn (S.A.); wangxmcn@scau.edu.cn (X.-M.W.); 2 School of Resources and Environment Sciences, Baoshan University, Baoshan 678000, China; bsxyxr2018@163.com; 3 Engineering Research Center of Biological Control, Ministry of Education, Guangzhou 510640, China; 4 Department of Entomology, South China Agricultural University, Guangzhou 510640, China; 5 Florida Department of Agriculture and Consumer Services, Division of Plant Industry, 1911 SW 34th Street, Gainesville, FL 32614-7100, USA; Muhammad.Ahmed@freshfromflorida.com; 6 Guangdong Laboratory of Lingnan Modern Agriculture, Guangzhou 510640, China

**Keywords:** biological control, *Cordyceps javanica*, *Eretmocerus hayati*, *Bemisia tabaci*, compatibility

## Abstract

Biological control is an effective method for whitefly management compared to the potential problems caused by chemical control, including environmental pollution and the development of resistance. Combined use of insect parasitoids and entomopathogenic fungi has shown high efficiency in *Bemisia tabaci* control. Here, we assessed the impacts of an entomopathogenic fungus, *Cordyceps javanica,* on the parasitism rate of a dominant whitefly parasitoid, *Eretmocerus hayati*, and for the first time also compared their separate and combined potential in the suppression of *B. tabaci* under semi-field conditions. Six conidial concentrations of *C. javanica* (1 × 10^3^, 1 × 10^4^, 1 × 10^5^, 1 × 10^6^, 1 × 10^7^ and 1 × 10^8^ conidia/mL) were used to assess its pathogenicity to the pupae and adults of *E. hayati*. Results showed that the mortality of *E. hayati* increased with higher concentrations of *C. javanica*, but these higher concentrations of fungus had low pathogenicity to both the *E. hayati* pupae (2.00–28.00% mortality) and adults (2.67–34.00% mortality) relative to their pathogenicity to *B. tabaci* nymphs (33.33–92.68%). Bioassay results indicated that *C. javanica* was harmless (LC_50_ = 3.91 × 10^10^) and slightly harmful (LC_50_ = 5.56 × 10^9^) to the pupae and adults of *E. hayati* respectively on the basis of IOBC criteria, and that *E. hayati* could parasitize all nymphal instars of *B. tabaci* that were pretreated with *C. javanica*, with its rate of parasitism being highest on second-instar nymphs (62.03%). Interestingly, the parasitoids from second and third-instar *B. tabaci* nymphs infected with *C. javanica* had progeny with increased longevity and developmental periods. Moreover, experimental data from 15 day semi-field studies indicate that combined application of *C. javanica* and *E. hayati* suppresses *B. tabaci* with higher efficiency than individual applications of both agents. Therefore, combined applications of *C. javanica* (1 × 10^8^ conidia/mL) and *E. hayati* is a more effective and compatible biological control strategy for management of *B. tabaci* than using either of them individually.

## 1. Introduction

The whitefly *Bemisia tabaci* (Gennadius) (Hemiptera: Aleyrodidae) is one of the most destructive insect pests of vegetables, ornamental plants and field crops worldwide [1]. Significant economic damage has been caused annually by this pest through its direct feeding damage to host plants, and indirectly by causing physiological disorders through honeydew secretion, leading to sooty mold accumulation on host plants. However, the most serious damage of this pest is that it can vector more than 110 species of plant viruses [1,2,3].

The silverleaf whitefly *B. tabaci* (also known as Middle East-Asia Minor 1 (MEAM1), formerly known as B biotype) initially invaded south China in the mid-1990s and successfully established its population due to its ability to reproduce quickly, develop resistance against commonly used chemicals and by having a wide host range [4]. The overuse of pesticides has increased selection pressure and expedited the development of resistance to multiple classes of chemical insecticides in different regions of the world [5,6,7,8]. Biological control methods are thus becoming a necessity for the control of *B. tabaci* to avoid the undesirable side effects of chemical control in the form of residues on crops, continuing chemical resistance development and pest resurgence [9,10,11,12,13,14].

Aphelinid parasitoids, such as the solitary *Eretmocerus hayati* (Rose and Zolnerowich) are some of the most important natural enemies of *B. tabaci*. Similar to other *Eretmocerus* parasitoids, *E. hayati* oviposits externally between the whitefly abdomen and the leaf surface of host plants [15]. After eclosion, the first instar penetrates the abdomen of the host whitefly nymph and develops internally [16]. It also feeds on the host haemolymph to obtain nutrients for living and egg development [17,18]. *Eretmocerus hayati* has shown high potential to be used as a biocontrol agent against *B. tabaci.* The original population of *E. hayati* was introduced into the USA from Pakistan in the 1980s and then brought to China by an Entomologist within the Chinese Academy of Sciences in 2008. In the following 10 years, its local populations increased and spread widely [9,18,19,20]. In the biological control of whitefly, Asad [21] suggested that when the average host densities *of B. tabaci* were about 10 nymphs/leaf in order to enhance whitefly suppression, alternative strategies may be required, for example, continuous low number release of parasitoids by banker plants to retain the parasitoids by non-host food, and that the release of *E. hayati* at 1:10 ratio (parasitoid/host) for three consecutive weeks could achieve efficient suppression of *B. tabaci*. Kuang et al. [22] demonstrated the potential for introducing *E. hayati* to control populations of *B. tabaci* on different host plants. At 26℃, the developmental period of *E. hayati* was 15.7, 16.1 and 15.2 d on *B. tabaci* infesting tomato, cucumber and collard plants, respectively; survivorship was 74.6%, 72.1% and 78.7%; longevity of a female adult was 7.4, 7.6 and 8.1 d; and the total numbers of eggs produced per female were 67.2, 62.3 and 74.7, respectively.

Entomopathogenic fungi have also been demonstrated to infect various types of insects [23,24,25,26,27] including *B. tabaci* [28,29,30]. Among the entomopathogenic fungi, *Cordyceps javanica* (Friederichs and Bally) (Hypocreales: Cordycipitaceae), formerly *Isaria javanica* (Friederichs and Bally) [31], is a widely used biological control agent of arthropod pests on vegetables, fruits, and ornamental plants [32,33,34,35], and several studies have demonstrated that *B. tabaci* is highly susceptible to this fungus [36,37]. For example, Xie et al. [38] reported that *B. tabaci* was susceptible to *C. javanica* by causing ≥90% mortality after 6 days by single-phase solid state fermentation on barley at 25 °C with 1 × 10^8^ conidia/g of initial substrate concentrations. 

Using a single biological product to suppress the population of whitefly may be difficult in practice. To this end, some previous studies have investigated the compatibility of entomopathogenic fungi and parasitoids and have evaluated the interactions between these two types of natural enemies as a potential biological control strategy for managing different types of insects, including coccids, aphids and whiteflies [39,40,41,42,43]. In order to improve the effectiveness of biological control of whitefly pests, it is imperative to know if a combination of two different biocontrol agents, such as an entomopathogenic fungus and an insect parasitoid, is more effective than their individual applications. This strategy may provide growers and landscape managers with an alternative option for whitefly management in agricultural ecosystems [42]. 

The objective of the current study was to evaluate the compatibility and efficacy of *E. hayati* and *C. javanica* in suppressing infestations of *B. tabaci*. The pathogenicity of *C. javanica* to the pupae and adults of *E. hayati* was analyzed through bioassays. Then *E. hayati* and *C. javanica* were released/applied simultaneously to observe their effect on *B. tabaci* populations under semi-field conditions.

## 2. Materials and Methods

### 2.1. Plants and Insects for Testing

Cotton plants (*Gossypium hirsutum* (Lumian 32)) at the 6–8 expanded leaf stage were cultured in plastic pots (15 cm diameter) containing a soil and sand mixture (10% sand, 5% clay and 85% peat) in a glasshouse at ambient temperature and photoperiod as described by Li et al. [44]. All experimental replications were performed on two symmetrical, fully expanded leaves with similar size. 

The *Bemisia tabaci* used in this study was the silverleaf whitefly, *B. tabaci* Middle East-Asia Minor 1 (*B. tabaci* B biotype). It was identified by using the mitochondrial COI sequence as described by Ahmed et al. [45]. Specimens of the parasitoid *E. hayati* were initially collected from the Institute of Plant Protection, Chinese Academy of Agricultural Sciences in 2015. Both the whitefly and parasitoid populations were maintained under laboratory conditions (26 ± 1 °C, 60% RH, L:D = 14:10) on cotton plants at South China Agricultural University (SCAU), Guangzhou.

### 2.2. Entomopathogenic Fungus

An entomopathogenic fungal strain of *C. javanica*, GZQ-1, was isolated from an adult Asian citrus psyllid cadaver in a glasshouse at SCAU, and the purified strain was deposited at the Guangdong Microbial Culture Collection Center (GDMCC) with the deposition number GDMCC 60437 [33].

### 2.3. Pathogenicity of C. javanica to E. hayati Pupae

Approximately 60 couples (60 male and 60 female adults) of *B. tabaci* adults were released into a cage (d × h = 3.0 × 1.5 cm, with a 100 mesh/cm^2^ cover at the top) on a single cotton leaf. Following 24 h for oviposition they were recaptured. The *B. tabaci* eggs (around 150–200 eggs per leaf) were allowed to hatch and develop to 2nd instar nymphs, after which 30 couples of 3 day old *E. hayati* adults were released into the leaf cage for 24 h and allowed to parasitize the whitefly nymphs (around 100 nymphs per leaf). When the *E. hayati* wasps reached the pupal stage in the *B. tabaci* nymphs (about 7 days after oviposition), these parasitized *B. tabaci* nymphs were dipped into six concentrations of *C. javanica* conidial suspensions (1 × 10^3^, 1 × 10^4^, 1 × 10^5^, 1 × 10^6^, 1 × 10^7^ and 1 × 10^8^ conidia/mL in 1% Tween-80 and ddH_2_O) for 15 s following the method of Cuthbertson et al. [46]. *E. hayati* nymphs sprayed with a solution of 1% Tween-80 in ddH_2_O acted as a negative control. Mortality rates of *E. hayati* pupae were investigated over the next 7 days (parasitoids that did not eclose 7 days after pupae stage were assumed to have died). All experiments were performed in an incubator (26 ± 1 °C, 90% RH and photoperiod 14:10 h L:D), and the bioassay for each concentration of *C. javanica* was replicated three times. 

### 2.4. Pathogenicity of C. javanica to E. hayati Adults

Cotton leaves with 2nd instar nymphs of *B. tabaci* (approximately 100 nymphs/leaf) were prepared using the same protocol described above and the cotton leaves were again dipped into six concentrations of *C. javanica* conidial suspensions (1 × 10^3^, 1 × 10^4^, 1 × 10^5^, 1 × 10^6^, 1 × 10^7^ and 1 × 10^8^ conidia/mL) for 15 s. Following this the leaves were allowed to air-dry and were then covered with leaf cages into which 30 pairs of 3 day old *E. hayati* adults were released in order to parasitize the nymphs. The mortality rates of *E. hayati* adults were investigated over the following 7 days. All experiments were performed in an incubator (26 ± 1 °C, 90% RH and photoperiod 14:10 h L:D). *E. hayati* adults sprayed with a 1% Tween-80 in ddH_2_O were used as control. The bioassay for each concentration of *C. javanica* was replicated three times. 

### 2.5. Effects of C. javanica on the Parasitic Potential of E. hayati

#### 2.5.1. No-Choice Test (Single Age of Whitefly Nymphs) 

Cotton leaves were prepared as previously described. Approximately 40 pairs of *B. tabaci* adults were released into one leaf cage to oviposit for 24 h. Following the timings in the methods of Cuthbertson et al. [30] and Qiu et al. [47], 1st to 4th instar nymphs were made available in different separate leaf cages for the same treatment date. Following this, the leaves that carried *B. tabaci* nymphs were sprayed with a 1 × 10^8^ conidia/mL suspension of *C. javanica* and subsequently air-dried. Then 30 pairs of *E. hayati* adults were released into each leaf cage and allowed to parasitize the nymphs for 24 h. The parasitism, emergence rate, developmental duration and longevity of the parasitoid were examined and recorded daily under a binocular microscope, after 7 days of parasitization until all the parasitoids or whiteflies had emerged. A 1% Tween-80 in ddH_2_O was used as a control and the experiment was replicated five times for all four instars of *B. tabaci* nymphs.

#### 2.5.2. Choice Test (Different Ages of Whitefly Nymphs)

Cotton plants and clean leaves were again prepared as outlined above. Ten couples of whitefly adults were released into a single leaf cage at four different times (total of 40 whitefly couples) separated by intervals between exposures. The intervals were as follows: T_0_ day (for first instar), plus T_2.5_ days (second instar), plus T_3_ days (third instar) plus T_2_ days (fourth instar) [45]. As with the no choice experiment, the leaves with *B. tabaci* nymphs were sprayed with 1 × 10^8^ conidia/mL suspension of *C. javanica*, and then air-dried after which 30 pairs of *E. hayati* adults were released into the leaf cage to parasitize for 24 h. The parasitism, emergence rate, developmental duration and longevity of parasitoid progeny were investigated as above. Parasitoids exposed to the silverleaf whitefly nymphs that were sprayed with 1% Tween-80 in ddH_2_O were used as controls. The experiments were replicated five times for all four instars of *B. tabaci* nymphs. 

### 2.6. Compatibility of C. javanica and E. hayati against B. tabaci 

The bioassay of *C. javanica* and *E. hayati* for the suppression of *B. tabaci* populations was carried out under semi-field conditions (cage in field) in April 2018 at SCAU (average temperature 24–30 °C, 50–60% RH). Cotton leaves with approximately 100 individuals/leaf of *B. tabaci* nymphs (with a mix of all four instars) were covered in leaf cages and treated by three methods: (1) Sprayed with 1 × 10^8^ conidia/mL suspension of *C. javanica*; (2) Exposed to thirty pairs of 3 day old *E. hayati* adults for 24 h; (3) Sprayed with the 1 × 10^8^ conidia/mL suspension of *C. javanica*, and then exposed to thirty pairs of 3 day old *E. hayati* adults for 24 h. Control leaves were sprayed with 1% Tween-80 in ddH_2_O. Each experimental treatment was repeated four times.

### 2.7. Statistical Analysis 

The infected parasitoid pupae and adults by the six conidial concentrations of *C. javanica* were observed daily. The average mortalities of parasitoid pupae and adults caused by the tested fungi were calculated. The mathematical calculation of lethal concentrations LC_50_ and confidence limits were carried out by the method of probit analysis [41]. Data regarding the mortality of *E. hayati* and *B. tabaci*, the parasitism, developmental time, emergence rate and longevity of *E. hayati* in treated vs. control experiments were subjected to t-test at *p* < 0.05. In the no-choice experiments (and the choice experiments), the parasitism, developmental time, emergence rate and longevity of *E. hayati* in different treatments was analyzed using main effect (and simple effect) of two-way ANOVA (analysis of variance) at *p* < 0.05 with the concentration of *C. javanica* suspension or *B. tabaci* instars as fixed factors. Treatment means were compared by Tukey’s HSD test (*p* < 0.05) after being arcsine square root transformed. All statistical analysis was performed using SPSS 19.0 (SPSS Inc., Chicago, IL, USA).

## 3. Results

### 3.1. Pathogenicity of C. javanica to E. hayati Pupae and Adults

**Figure 1:**
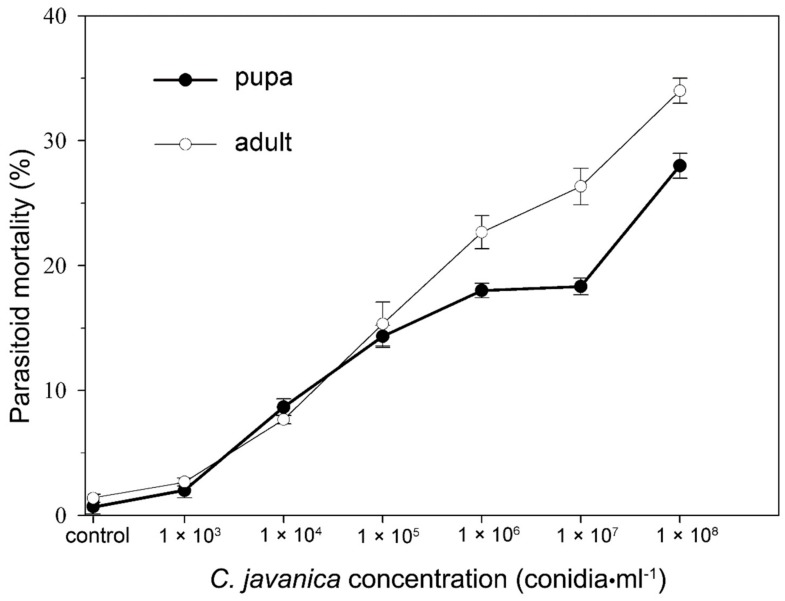
The mortality of *Eretmocerus hayati* pupae and adults when treated with various concentrations of *Cordyceps javanica* (1 × 10^3^ to 1 × 10^7^ conidia/mL). Data are Mean ± SE of 3 replicates.

*Cordyceps javanica* had low pathogenicity against the pupa and adults of *E. hayati*. After 7 days following application, the mortality rates of the *E. hayati* pupa were 2.00 ± 0.58%, 8.67 ± 0.66%, 14.33 ± 0.88%, 18.00 ± 0.58%, 18.33 ± 0.67% and 28.00 ± 1.00% for 1 × 10^3^, 1 × 10^4^, 1 × 10^5^, 1 × 10^6^, 1 × 10^7^ and 1 × 10^8^ conidia/mL, respectively, whereas the adult mortalities were 2.67 ± 0.33%, 7.67 ± 0.33%, 15.33 ± 1.76%, 22.67 ± 1.33%, 26.33 ± 1.45% and 34.00 ± 1.00% for the same series of concentrations of *C. javanica* (Figure 1). The LC_50_ values for *C. javanica* against the pupae and adults of *E. hayati* were 3.91 × 10^10^ and 5.56 × 10^9^ conidia/mL (Table 1). Meanwhile, the concentration of *C. javanica* was positively correlated to the pathogenicity against *E. hayati*.

### 3.2. Effects of C. javanica on E. hayati Wasp’s Parasitic Potential

**Figure 2:**
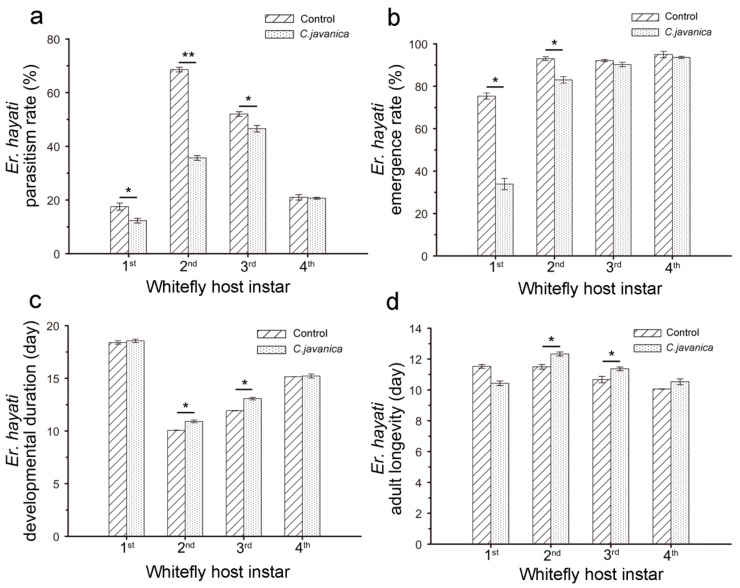
The parasitic potential of *Eretmocerus hayati* against *Bemisia tabaci* nymphs pretreated with *Cordyceps javanica* (1 × 10^8^ conidia/mL) in the non-selective test. Error bars represent Mean ± SE. The * and ** indicate significant difference at *p* < 0.05 and *p* < 0.01 level; main effect of two-way ANOVA (analysis of variance).

Significant differences were observed in the rate of parasitism, emergence rate, longevity and developmental duration of *E. hayati* when parasitizing young nymphs of *B. tabaci* that were pretreated with *C. javanica.* In the no-choice experiment (single age of whitefly nymphs), the parasitism rate and emergence rate of *E. hayati* to pretreated *B. tabaci* nymphs was reduced, especially against the 1st and 2nd instar nymphs of *B. tabaci* (Figure 2a,b). This was particularly evident in the longevity and developmental duration of *E. hayati* on 2nd and 3rd instar nymphs, which were significantly higher than that in the control (Figure 2c,d).

**Figure 3:**
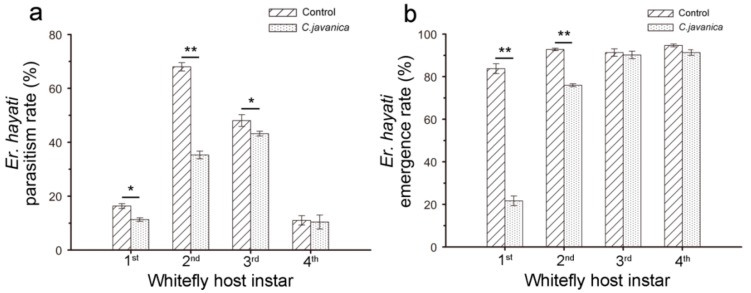
The parasitic potential of *Eretmocerus hayati* against *Bemisia tabaci* nymphs pretreated with *Cordyceps javanica* (1 × 10^8^ conidia·ml^−1^) in the choice test (multi-age). The * and ** indicate significant difference at *p* < 0.05 and *p* < 0.01 level; simple effect of two-way ANOVA (analysis of variance).

In the choice experiment (different ages of whitefly nymphs)**,** under the interaction of treatments and *B. tabaci* instar, the parasitism rate and emergence rate of *E. hayati* reared on 1st and 2nd instar nymphs were especially reduced when all four instars of whitefly nymphs were available (Figure 3a,b).

### 3.3. Compatibility of C. javanica and E. hayati against B. tabaci 

**Figure 4:**
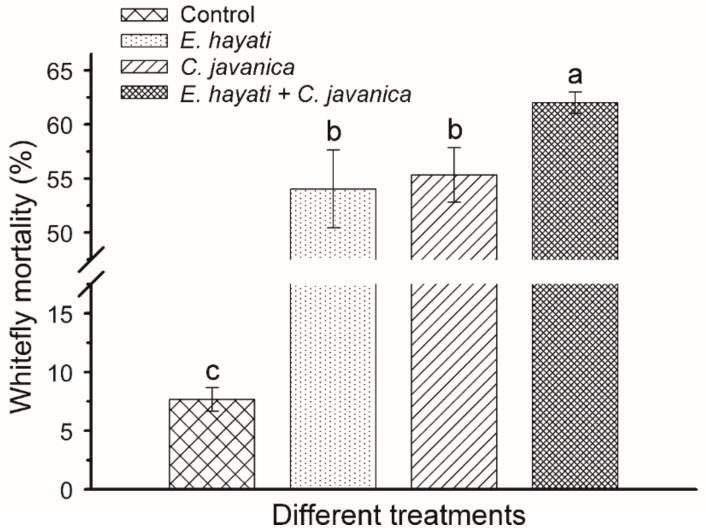
Separate and combined applications of *Cordyceps javanica* (1 × 10^8^ conidia/mL) and *Eretmocerus hayati* against *Bemisia tabaci*. Dates are the Mean ± SE after 15 days of spray or release under semi-field conditions. The different letters over the bars indicate significant differences between the four treatments (*p* < 0.05).

The results of semi-field experiments provided evidence that the combination of *C. javanica* and *E. hayati* was more effective than individual separate applications for the suppression of *B. tabaci* populations (Figure 4). After 15 days of investigation, the whitefly mortality rates were significantly different among the three applications (F = 363.96, *p* < 0.05), 55.33%, 54.03% and 62% for *C. javanica*, *E. hayati* and *C. javanica* + *E. hayati*, respectively. 

## 4. Discussion

The compatibility of two different types of biological control regents is very important for sustained and successful pest management. Our bioassay results indicated that *C. javanica* was harmless (1, IOBC category) and slightly harmful (2, IOBC category) to the pupae (2.0–28.0%) and adults (2.67–34%) respectively of *E. hayati* on the basis of IOBC criteria [48]. The results in the current study indicate that both biocontrol agents (*C. javanica* and *E. hayati*) are compatible when applied together for *B. tabaci* management and as a result provide better control than separate applications of the two agents. These results are consistent with other studies [49,50]. 

The entomopathogenic fungi may infect an insect natural enemy of the same pest. Our study demonstrated that *C. javanica* had low pathogenicity to *E. hayati* pupae and adults. Ibarra-Cortes et al. [51] reported that *Diaphorina citri* Kuwayama nymphs and their hymenopteran parasitoid, *Tamarixia radiata* (Waterston), were more susceptible to fungal isolates than *D. citri* adults. Moreover, multiple studies suggest that fungal sprays did not affect natural enemies and posed a negligible risk to their behaviour [52,53,54]. Indeed, Huang et al. [55] showed a non-significant effect of the fungus *Isaria fumosoroseus* on longevity and next offspring of females of the parasitoid *E. furuhashii* Rose and Zolnerowich. Although different pathogenic species or strains of fungi have different pathogenicities and virulences, the target effect of a fungus can be quite specific to one or two hosts [27]. As such, additional studies are necessary to identify interactions between microbes and insect natural enemies to further develop biological pest control programs.

It is important to assess the interactions between biological control agents in the field. Our study provides the data and basis for designing a biocontrol system that simultaneously utilizes two biocontrol agents, which may offer effective substitutes for chemical pesticides for the control of *B. tabaci*. However, the compatibility of the two agents and environmental effects on the efficacy of each agent alone and in combination for managing *B. tabaci* needs to be further evaluated. It is evident from our findings that there are positive interactions between the two different biocontrol agents, and these interactions could serve as an effective control tool.

## 5. Conclusions

Biological control is currently considered an effective alternative method for whitefly management. Joint application of different biocontrol regents together, such as parasitoids and entomopathogenic fungi, may have higher efficiency than using one agent individually. In the current study, we assessed the impacts of *C. javanica* on the biology of the parasitoid *E. hayati*, and compared their separate and combined potential for the suppression of *B. tabaci* under both laboratory and semi-field conditions. The findings of our study are expected to improve the efficiency of whitefly pest management in agricultural ecosystems.

## Figures and Tables

**Table 1:** The pathogenicity evaluation of *Cordyceps javanica* (LC_50_) to the pupae and adults of *Eretmocerus hayati* in the first 7 days following application.

Insect Stages	Regression Virulence Model	LC_50_ and 95% CI (conidia/mL)	R^2^
Pupa	Y = 0.219X – 2.316	3.91 (0.55~72.2) × 10^10^	0.931
Adult	Y = 0.241X – 2.350	5.56 (1.27~44.6) × 10^9^	0.963
